# CYP4B1 inhibits lung adenocarcinoma progression via PI3K/AKT/mTOR pathway: mechanistic insights and development of a CYP4B1-related prognostic signature

**DOI:** 10.3389/fonc.2025.1661650

**Published:** 2026-01-26

**Authors:** Guoyin Li, Jing Kang, Xiaoyan Li, Liping Zhao, Yuhang Chen, Chenlu Gong, Zhiqiang Liu, Bi Zhang

**Affiliations:** 1 College of Life Science and Agronomy, Zhoukou Normal University, Zhoukou, China; 2 Key Laboratory of Modern Teaching Technology, Ministry of Education, Shaanxi Normal University, Xi’an, China; 3 Department of Clinical Laboratory, Shanxi Bethune Hospital, Shanxi Academy of Medical Sciences, Tongji Shanxi Hospital, Third Hospital of Shanxi Medical University, Taiyuan, China; 4 Department of Blood Transfusion, Shanxi Provincial People’s Hospital, Taiyuan, Shanxi, China; 5 Department of Pathology, Shanxi Provincial People’s Hospital, Taiyuan, Shanxi, China

**Keywords:** CYP4B1, lung adenocarcinoma, NFIA, PI3K/AKT/mTOR, prognostic model

## Abstract

**Background:**

Lung adenocarcinoma (LUAD) has a poor 5-year survival rate due to delayed diagnosis and drug resistance. Cytochrome P450 4B1 (CYP4B1), a lung-predominant enzyme, is linked to cancer susceptibility, but its role and regulation in LUAD remain unclear.

**Methods:**

Multi-omics analyses of public datasets (TCGA_LUAD, GSE series) and clinical specimens assessed CYP4B1 expression. Functional experiments (cell lines, xenografts) explored its effects. Mechanistic studies (Western blot, ChIP) and transcriptional regulation assays (luciferase reporters) were performed. A CYP4B1-related risk score (CRRS) and nomogram were developed via LASSO-Cox regression and validated.

**Results:**

CYP4B1 is significantly downregulated in LUAD, correlating with poor patient survival (AUC > 0.78 for diagnostic discrimination between LUAD and normal tissues). Functionally, CYP4B1 inhibits LUAD cell proliferation *in vitro* and *in vivo*, which is associated with the suppression of the PI3K/AKT/mTOR signaling pathway. We identified a transcriptional regulatory mechanism in which transcription factor nuclear factor I A (NFIA) acts as a key upstream regulator of CYP4B1, activating its transcription via direct binding to the CYP4B1 promoter. The CRRS, constructed based on 7 CYP4B1-related core genes, effectively stratified patients into high/low-risk groups with divergent survival outcomes in both training and validation cohorts, and correlated with drug sensitivity. A nomogram integrating CRRS and clinical variables achieved reliable prediction of 1-/3-/5-year survival (AUC > 0.72).

**Conclusion:**

CYP4B1 functions as a tumor suppressor in LUAD, regulated by NFIA and exerting its effects through PI3K/AKT/mTOR pathway inhibition. The CRRS and nomogram provide potential tools for prognosis assessment and offer guidance for precision therapy in LUAD, pending further translational validation.

## 1 Introduction

Lung cancer is the second most common malignancy and the leading cause of cancer-related deaths worldwide (1). Lung adenocarcinoma (LUAD) is the predominant subtype, accounting for approximately 40% of all lung cancer cases (2). Unfortunately, the 5-year survival rate of patients with LUAD is less than 20%, which remains discouraging. This statistic is largely due to delayed diagnosis, frequent recurrences, and increasing drug resistance (3). Despite advances in diagnostic and therapeutic strategies, the survival outcomes for patients with LUAD have not improved significantly. Hence, a deeper understanding of the molecular mechanisms driving this disease and the development of individualized prognostic models are urgently needed to enhance precision therapy.

Cytochrome P450 monooxygenases (P450s) are a class of heme-thiolate proteins with a broad distribution across living organisms (4). Their primary function lies in catalyzing the reductive cleavage of molecular oxygen (5). In animals, P450s are predominantly localized in the hepatic endoplasmic reticulum, while also present in tissues like mitochondria, intestines, and lungs. These enzymes not only participate in the synthesis and metabolism of endogenous substances (6, 7) to sustain homeostasis but also act as a key enzymatic system in the “biotransformation” of drugs within the body (8).

CYP4B1, a member of the CYP4 family, differs from other family members by functioning at the interface of endogenous and exogenous metabolism. It can hydroxylate common endogenous substrates such as fatty acids and activate exogenous drugs including 4-ipomeanol, perilla ketone, and valproic acid (9–11). CYP4B1 mainly catalyzes the ω-, ω-1-, and subterminal (α-, β-, γ-, δ-) hydroxylation of fatty acids, exhibiting particularly high efficiency toward medium-chain fatty acids (C9-C16) (12). Compared with other members of the CYP4 family (e.g., CYP4A, CYP4F), the uniqueness of CYP4B1 lies in its multi-positional hydroxylation capability and its preference for unsaturated fatty acids. CYP4B1 can metabolize arachidonic acid into 20-HETE, which can increase NADPH oxidase-derived reactive oxygen species (ROS) production (13, 14). CYP4B1 is mainly expressed in the lung (15), with detectable levels also found in adipose tissue, bladder, esophagus, and stomach (16). Previous studies have indicated that CYP4B1 polymorphisms are significantly associated with the risk of various cancers, including gastric cancer (16), bladder cancer (17), and lung cancer (18). Specifically in the context of LUAD, previous studies have reported the downregulation of CYP4B1 mRNA in tumor tissues compared to normal lung (19), and bioinformatic analyses have suggested its potential as a prognostic biomarker (20). However, a comprehensive mechanistic understanding of its tumor-suppressive role, including its upstream regulation and downstream signaling pathways, has remained elusive. Our study aims to fill this critical knowledge gap by: 1) elucidating the upstream transcriptional regulation of CYP4B1 by the transcription factor NFIA; 2) defining the functional link between CYP4B1 and the oncogenic PI3K/AKT/mTOR signaling pathway; and 3) developing and validating a clinically applicable CYP4B1-related prognostic signature (CRRS).

NFIA is a transcription factor involved in organ development and cellular differentiation. Notably, emerging evidence suggests a tumor-suppressive role for NFIA in NSCLC, where its expression is frequently downregulated, and it has been shown to inhibit oncogenic signaling pathways (21, 22). Given its known function as a transcriptional regulator and its relevance in lung cancer, we hypothesized that NFIA might be an upstream regulator responsible for modulating CYP4B1 expression in LUAD. In this study, we observed that CYP4B1 is abnormally downregulated in LUAD, and this low expression is closely linked to poor patient prognosis. Additionally, both *in vitro* and *in vivo* investigations revealed that CYP4B1 can inhibit the proliferation of LUAD cells by suppressing the PI3K/AKT/mTOR signaling pathway. The transcription of CYP4B1 is directly regulated by NFIA. Furthermore, leveraging insights from the CYP4B1-related regulatory network, we developed and validated a prognostic signature to assess its clinical translational potential. We acknowledge that the primary datasets used (TCGA and GEO) may have inherent population-specific biases; therefore, our analytical approach and model construction incorporated multiple independent cohorts to enhance the robustness and generalizability of our findings.

## 2 Materials and methods

### 2.1 Public data acquisition and processing

The TCGA_LUAD dataset was obtained from The Cancer Genome Atlas (TCGA) database (https://portal.gdc.cancer.gov). Additionally, the datasets GSE72094, GSE11969, GSE26939, GSE41271, GSE31210, GSE10072, GSE46539, and GSE32863 were downloaded from the Gene Expression Omnibus database (https://ncbi.nlm.nih.gov/gds). All patients included in this study were diagnosed with lung adenocarcinoma, and the datasets used contained detailed clinical information. All datasets were processed using the methodologies previously outlined in our studies (23, 24). Ethical approval was not required since the datasets were publicly available.

### 2.2 Unsupervised clustering

Consensus clustering (CC) was performed using the ‘ConsensusClusterPlus’ package in R 4.1.0 with the following parameters: 80% item resampling, 80% gene resampling, 1000 repetitions, and Pearson correlation as the distance metric. Nonnegative matrix factorization (NMF) clustering was conducted using the ‘NMF’ package with the’brunet’algorithm and 100 iterations (25).

### 2.3 Development of CRRS

The TCGA_LUAD cohort was used as the training set for CRRS development, whereas the datasets GSE72094, GSE41271, and GSE31210 served as validation sets.

The 42 genes co-expressed with CYP4B1 (**Supplementary Table 1**; correlation coefficient 0.5; *P* ≤ 0.001) was identified from the TCGA_LUAD dataset. Based on the expression levels of CYP4B1 and its co-expressed genes, and integrating CC and NMF, we stratified the patients in the TCGA_LUAD dataset into two subgroups and identified 38 differentially expressed genes (DEGs) that were significantly associated with prognosis (**Supplementary Table 2**; |logFC| ≥ 2; Adjusted *P* value ≤ 0.01). The Least Absolute Shrinkage and Selection Operator (LASSO) Cox regression model was applied to these genes, yielding seven key genes and their coefficients. A risk score for each patient was calculated using the following formula: score = ∑_i_ Coefficient(Gene i) × Expression(Gene i) (26).

### 2.4 Drug sensitivity analysis

The drug sensitivity analyses were conducted using the “limma” and “pRRophetic” packages in R 4.1.0, considering *P* < 0.001 and setting the seed to 12345.

### 2.5 Development and evaluation of the nomogram

A nomogram was developed to quantify patient prognosis based on CRRS, gender, age, tumor stage, T stage, and N stage. This was constructed using the “survival,” “survminer,” “timeROC,” “rms,” and “regplot” packages in R 4.1.0.

### 2.6 Cell culture and lentiviral infection

Human LUAD cell lines H1299 and PC-9 were obtained from the Cell Bank of the Shanghai Institute of Biological Sciences, Chinese Academy of Sciences. The PC-9 and H1299 human LUAD cell lines were selected to represent distinct genetic backgrounds relevant to LUAD pathogenesis. PC-9 cells harbor an EGFR exon 19 deletion mutation, which is a common driver in LUAD, while H1299 cells are p53-null, representing another frequent genetic alteration. Using these two models strengthens the generalizability of our findings across different LUAD genomic contexts. CYP4B1 knockdown/overexpression lentiviruses were purchased from Shanghai Jikai Biotechnology Co., Ltd. For infection, cells were seeded in 6-well plates and at approximately 60% confluence, were incubated with the lentiviral particles and 8 μg/mL Polybrene for 24 hours. Subsequently, the medium was replaced with fresh complete medium. Stable cell pools were selected using 2 μg/mL puromycin for at least one week.

### 2.7 Colony formation assay

LUAD cells were seeded into 6-well plates at a density of 500 cells per well and cultured for 2 weeks. The cells were subsequently fixed with cold methanol and stained with crystal violet for colony visualization.

### 2.8 Patients and specimens

The LUAD samples were obtained from the Pathology Department of Shanxi Provincial People’s Hospital (Taiyuan, China). The use of clinical specimens was authorized by the ethics committee of Shanxi Provincial People’s Hospital (2021-196). All procedures were carried out in accordance with relevant guidelines and regulations. All experimental protocols were approved by the ethics committee of Shanxi Provincial People’s Hospital. The informed consent was obtained from all participants and/or their legal guardian(s).

### 2.9 Immunohistochemistry and immunofluorescence assays

LUAD and adjacent noncancer tissues were processed into tissue microarrays. The slides were stained with immunohistochemistry (IHC), scanned using a Panoramic MIDI (3DHISTECH, Ltd., Budapest, Hungary), and analyzed with the Panoramic Viewer v. 1.15.3 and Nuclear Quant application for PV v.2.0.0.46136 (3DHISTECH). The H-score was calculated to quantify the protein levels of target genes in the tissues, using the formula: H-score = ∑ (*pi* × *i*) =(percentage of weak intensity × 1) + (percentage of moderate intensity × 2) + (percentage of strong intensity × 3), where (*i*) denotes the intensity of staining, and (*pi*) is the percentage of stained tumor cells (27). For immunofluorescence (IF), cells were seeded on confocal dishes. Upon reaching ~60% confluence, cells were fixed with 4% paraformaldehyde for 15 min, permeabilized with 0.3% Triton X-100 for 10 min, and blocked with 5% bovine serum albumin (BSA) for 1 hour at room temperature. Cells were then incubated with primary antibodies overnight at 4 °C, followed by incubation with fluorochrome-conjugated secondary antibodies for 1 hour at room temperature in the dark. Nuclei were counterstained with DAPI. Images were captured using a Zeiss LSM 900 confocal laser scanning microscope. Information on the antibodies used for IHC and IF is provided in **Supplementary Table 3**. For IHC/IF, negative controls were performed by omitting the primary antibody, resulting in no specific staining. Staining of adjacent normal lung tissue served as the positive internal control for CYP4B1.

### 2.10 Cell cycle assay

The cells were plated in six-well plates and subjected to a starvation treatment for 12 h in a medium supplemented with 1% FBS. Following the starvation, the cells were cultured for an additional 24 h in a complete culture medium. Subsequently, the cell cycle distribution was assessed using a cell cycle assay kit (AC12L553, Life-iLab, China) and analyzed by flow cytometry. The cell cycle populations were gated based on DNA content (PI fluorescence) after excluding doublets and debris. Representative gating strategies is provided in **Supplementary Figure 3**.

### 2.11 EdU staining

The cells were seeded in laser confocal dishes and subjected to a starvation treatment for 12 h using a medium supplemented with 1% FBS. Following this, the cells were cultured in a complete medium for an additional 24 h. The cells were treated with BeyoClick EdU-555 (C0075L, Beyotime, China) to assess cell proliferation and subsequently labeled with DAPI for nuclear visualization. The cells were detected using a confocal laser scanning microscope.

### 2.12 CCK-8 assay

Cells were seeded in 96-well plates at a density of 2,000 cells per well. At the indicated time points (0, 24, 48, 72 hours), 10 μL of CCK-8 reagent (AC11L054, Life-iLab) was added to each well and incubated for 1 hours at 37 °C. The absorbance at 450 nm was measured using a microplate reader (BioTek, USA). Each experiment was performed with at least four replicate wells per group.

### 2.13 Wound healing assay

Cells in the logarithmic growth phase (PC-9 and H1299) were digested with 0.25% trypsin. After centrifugation at 1000 rpm for 5 minutes, the cells were resuspended in complete medium, counted, and adjusted to a concentration of 3×10^5^ cells/mL. 2 mL of the cell suspension was added to each well of a 6-well plate, gently shaken to ensure uniform distribution of the cells, and then placed in an incubator for 24–48 hours until the cell confluency reached 80%-90%. 1straight line were scratched in the center of the cell monolayer in each well using 200μL sterile pipette tips, with the tips held perpendicular to the wall of the 6-well plate. Subsequently, the wells were rinsed twice with sterile PBS. 2 mL of serum-free medium was added to each well. Under an inverted phase-contrast microscope, 3 fixed fields of view in the scratched area were selected, and images were captured and recorded as 0 h data. After continuous culture in the incubator for 48 hours, images were captured again. The scratch width was measured, and the cell migration rate was calculated using the formula: Migration rate (%) = (Scratch width at 0 h - Scratch width at 48 h)/Scratch width at 0 h × 100%. Each experimental group was set up with 3 replicate wells. To assess collective cell migration, assays were performed in serum-free medium without the use of proliferation inhibitors, acknowledging that the measured “migration rate” may incorporate a component of proliferation at the wound edge.

### 2.14 Transwell assay

Matrigel Dilution: Dilute Matrigel with pre-chilled serum-free medium at a ratio of 1:8 (operation on ice), and the diluted Matrigel must be used within 30 minutes. Matrigel Coating: Add 80 μL of diluted Matrigel to the upper chamber of the Transwell insert, ensuring it evenly covers the entire surface of the membrane. Gelation: Place the Transwell insert with coated Matrigel into a 37 °C incubator with 5% CO_2_, and incubate for 2–4 hours to allow the Matrigel to polymerize into a solid state. Hydration: Remove the insert from the incubator and carefully aspirate the excess liquid (uncoagulated) from the upper chamber. Add 300 μL of serum-free medium to the upper chamber and 500 μL of complete medium to the lower chamber. Place the insert back into the incubator for hydration for 1–2 hours to balance the environment inside and outside the membrane. After hydration, aspirate the medium from both the upper and lower chambers. Cell Preparation: Digest cells (PC-9 and H1299) in the logarithmic growth phase with trypsin. Resuspend the cells in serum-free medium, then centrifuge and wash them 1–2 times to remove serum. Resuspend the cells again in serum-free medium, count the cells, and adjust the cell density to 2×10^5^ cells/mL. Lower Chamber Liquid Addition: Add 600 μL of complete medium containing 10% FBS to the lower chamber. Upper Chamber Seeding: Carefully add 100 μL of the cell suspension to the upper chamber of the Transwell insert. Culture: Gently place the culture plate into a 37 °C incubator with 5% CO_2_ and incubate for 48 hours. Experiment Termination: Carefully remove the Transwell insert and gently rinse it twice with PBS. Fixation: Place the insert into a well containing 4% paraformaldehyde and fix at room temperature for 20–30 minutes. Washing: Take out the insert and gently rinse it 2–3 times with PBS. Staining: Place the insert into a well containing 0.1% crystal violet staining solution and stain at room temperature for 15–30 minutes. Washing Again: Gently rinse the insert with PBS several times until the eluate becomes colorless. Use a wet cotton swab to carefully wipe off the non-migrated cells on the surface of the membrane in the upper chamber. Air-Drying: Invert the insert and allow it to air-dry naturally. Photography and Counting: Photograph the insert and count the number of cells that have migrated through the membrane. Each group was set up with 3 duplicate wells.

### 2.15 qRT-PCR assay

Total RNA was extracted from cells using Trizol reagent (Life-iLab, AN51L758) according to the manufacturer’s protocol. Reverse transcription was performed with SuperRT III One-Step Reverse Transcription Kit (Biosharp, BL1020B), followed by cDNA amplification and detection using 2 x qPCR Mix(SYBR Green) (Life-iLab, AN19L918). GAPDH served as the internal loading control.

### 2.16 Dual-luciferase reporter gene assay

We cloned the wild-type sequence of 2000 bp upstream of the transcription start site of the human CYP4B1 gene, as well as sequences with mutations in single or multiple potential binding sites of NFIA, into the pGL3-Basic vector individually. The resulting plasmids were co-transfected into 293T cells alongside a Renilla luciferase expression construct. Forty-eight hours after transfection, the cells were harvested and cell lysates were prepared. Luciferase activity was subsequently determined using the Dual-Luciferase Reporter Assay Kit (YEASEN, 1142ES60).

### 2.17 ChIP assay

The ChIP assay was performed using the Beyotime ChIP Assay Kit (P2078, China) with PC-9 cells. The ChIP assay was performed strictly in accordance with the manufacturer’s protocol. After immunoprecipitation with NFIA antibody (CST, 69375) or normal rabbit IgG (CST, 2729), the enriched DNA was analyzed by qPCR using primers flanking the putative NFIA binding site on the CYP4B1 promoter and primers for a negative control region. Primer sequences were provided in **Supplementary Table 3**.

### 2.18 Western blot analysis

Total proteins were extracted from the cells using RIPA lysis buffer and quantified using a BCA protein quantification kit (AP12L025, Life-iLab). Equal amounts of protein (30 μg) were separated by SDS-PAGE and transferred onto NC membranes. The membranes were blocked with 5% non-fat milk in TBST for 1 hour and then incubated with primary antibodies overnight at 4 °C. After washing, membranes were incubated with HRP-conjugated secondary antibodies for 1 hour at room temperature. Protein bands were visualized using an Enhanced Chemiluminescence (ECL) substrate (AP34L024, Life-iLab) and imaged with a ChemiDoc Imaging System (5200, Tanon). GAPDH was used as a loading control. Information on the antibodies used for Western blotting is provided in **Supplementary Table 3**.

### 2.19 Xenograft LUAD model

Six-week-old female nude BALB/c mice were obtained from Beijing Vital River Laboratory Animal Technology Co., Ltd. The cells were mixed with a matrix adhesive in a 1:1 ratio and injected subcutaneously into the dorsal region of the mice, with a total of 5 × 10^6^ cells per mouse (five replicates per group). The tumor volumes were measured starting from day 8 post-injection, with measurements taken every 3 days until the mice were euthanized. Euthanasia was performed using the Animal Multi-functional Anesthesia Machine CO_2_ Euthanasia Device (HOPE-MED8160, HePu Industry and Trade Co., Ltd., Tianjin, China). Place the mice in the sealed chamber, open the CO_2_ flow valve (using 100% pure CO_2_ with the flow rate set to 20% of the container volume per minute), and continue introducing CO_2_ until the breathing stops, then maintain this for another 2 minutes to ensure thorough death. The animal research protocol was approved by the ethics committee of Shanxi Provincial People’s Hospital (2021-191). All methods were conducted in accordance with relevant guidelines and regulations, and all procedures adhered strictly to The Animal Research: Reporting of *In Vivo* Experiments (ARRIVE) guidelines.

### 2.20 Statistical analysis

The bioinformatics section utilized R software version 4.1.0 for statistical analysis, data evaluation, and visualization. The Wilcoxon rank-sum test is used to assess differences in the expression of the target gene between the two groups. The log-rank test is used to analyze differences in survival between the two groups of patients. Results of *in vitro* (n = 3) and *in vivo* (n = 5) experiments were plotted using GraphPad Prism 9, and Student’s t-test were applied to analyze differences between the two groups. The sample sizes for *in vitro* experiments were determined based on preliminary data and established practices in the field to ensure detectable effect sizes. For *in vivo* experiments, a sample size of n = 5 per group was initially chosen based on common practice in xenograft studies. A *P* value <0.05 indicated a statistically significant difference (^*^P < 0.05; ^**^P < 0.01; ^***^P < 0.001).

## 3 Results

### 3.1 Integrative multi-omics analysis reveals that CYP4B1 is a key tumor suppressor gene and prognostic indicator in LUAD

**Figure 1:**
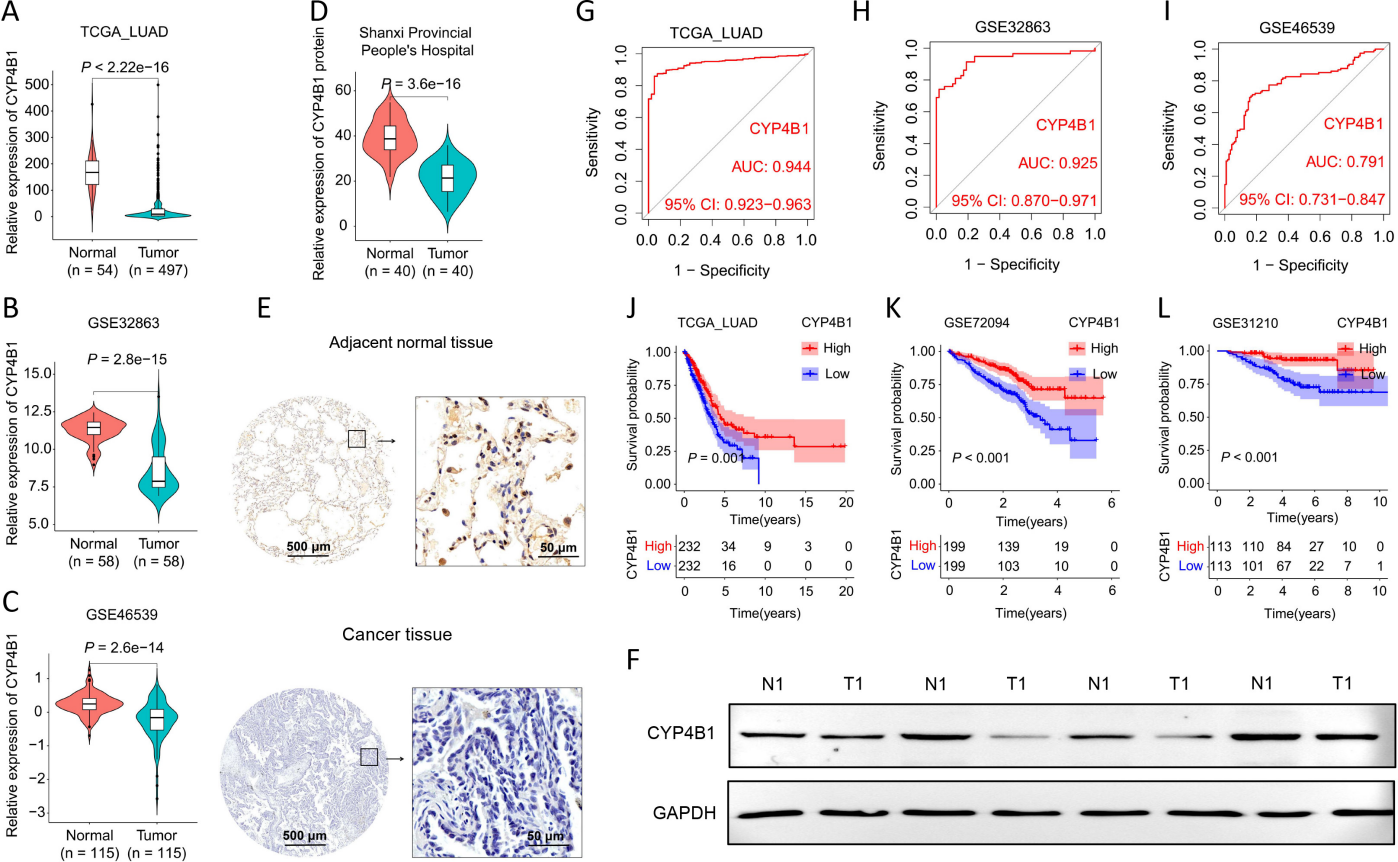
CYP4B1 downregulation is associated with LUAD malignancy and predicts poor clinical prognosis. **(A–C)** CYP4B1 mRNA expression levels in normal vs. LUAD tissues from TCGA_LUAD, GSE32863, and GSE46539 datasets. **(D–E)** IHC analysis of CYP4B1 protein expression in 40 paired LUAD and adjacent normal tissues from Shanxi Provincial People’s Hospital, including quantification and representative images. **(F)** WB validation of CYP4B1 protein levels in human LUAD vs. adjacent normal tissues. **(G–I)** ROC curve analysis of CYP4B1 expression for distinguishing LUAD from normal tissues in TCGA_LUAD, GSE32863, and GSE46539. **(J–L)** Kaplan-Meier survival curves for LUAD patients stratified by CYP4B1 expression in TCGA_LUAD, GSE72094, and GSE31210 datasets.

As an extrahepatic cytochrome P450 isoform, CYP4B1 is predominantly expressed in the lung, with lower expression levels in other organs (28). However, its expression patterns and functional mechanisms in LUAD remain incompletely understood, necessitating further investigation. Prior studies have implicated CYP4B1 as a tumor suppressor in LUAD (29). To characterize CYP4B1 expression in LUAD, we performed multi-dataset analyses across TCGA_LUAD, GSE32863, GSE46539, GSE43458, GSE10072, and GSE31210. These analyses revealed a significant downregulation of CYP4B1 mRNA in LUAD tissues compared to normal lung tissues (**Figures 1A–C**). Consistently, in clinical tissue samples, we detected a marked reduction in CYP4B1 protein expression in LUAD tissues relative to normal lung tissues (**Figures 1D, E**; **Supplementary Figure 1**), and Western blot (WB) assays further confirmed this downregulation in tumor tissues (**Figure 1F**). We next evaluated the diagnostic potential of CYP4B1. ROC curve analyses across the above six datasets showed area under the curve (AUC) values > 0.78, indicating robust discriminatory power for distinguishing LUAD from normal tissues (**Figures 1G–I**; **Supplementary Figure 2**). Survival analyses in three independent datasets (TCGA_LUAD, GSE72094, GSE31210) further demonstrated that higher CYP4B1 expression correlated with better patient prognosis (**Figures 1J–L**). Collectively, our integrative multi-omics analyses validate that CYP4B1 downregulation serves as a molecular hallmark of LUAD pathogenesis and a clinically actionable prognostic biomarker.

### 3.2 CYP4B1 inhibits the proliferation of LUAD cells by suppressing the PI3K/AKT/mTOR signaling pathway

**Figure 2:**
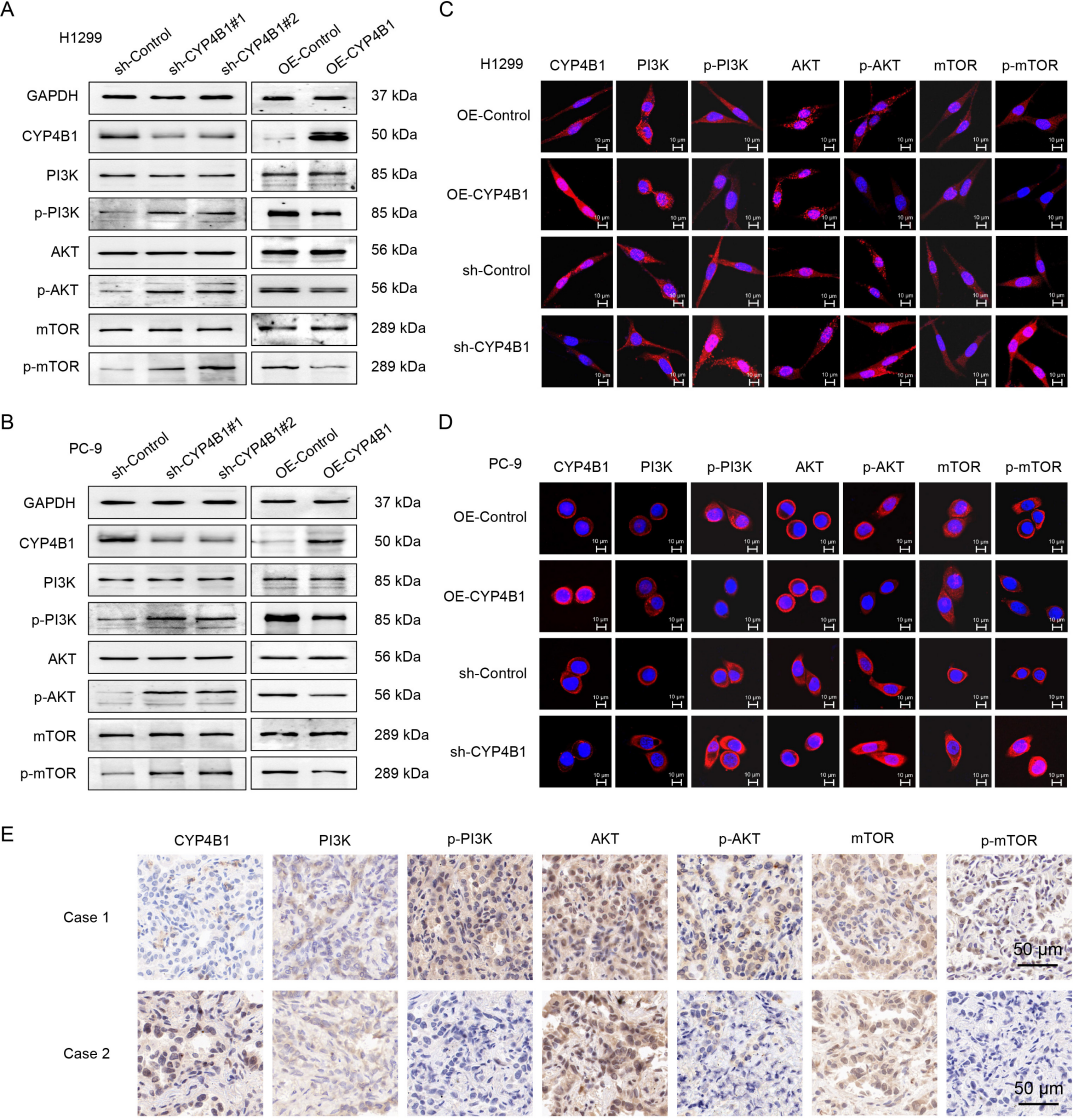
CYP4B1 inhibits the PI3K/AKT/mTOR signaling pathway. **(A, B)** After overexpressing/knocking down CYP4B1 in PC-9 and H1299 cells, the expression levels of the target proteins were detected by WB. **(C, D)** Overexpression/knockdown of CYP4B1 in PC-9 and H1299 cells and detection of the expression level of target protein by immunofluorescence assay. **(E)** IHC assay was performed to detect the expression level of the target protein in human LUAD tissues.

**Figure 3:**
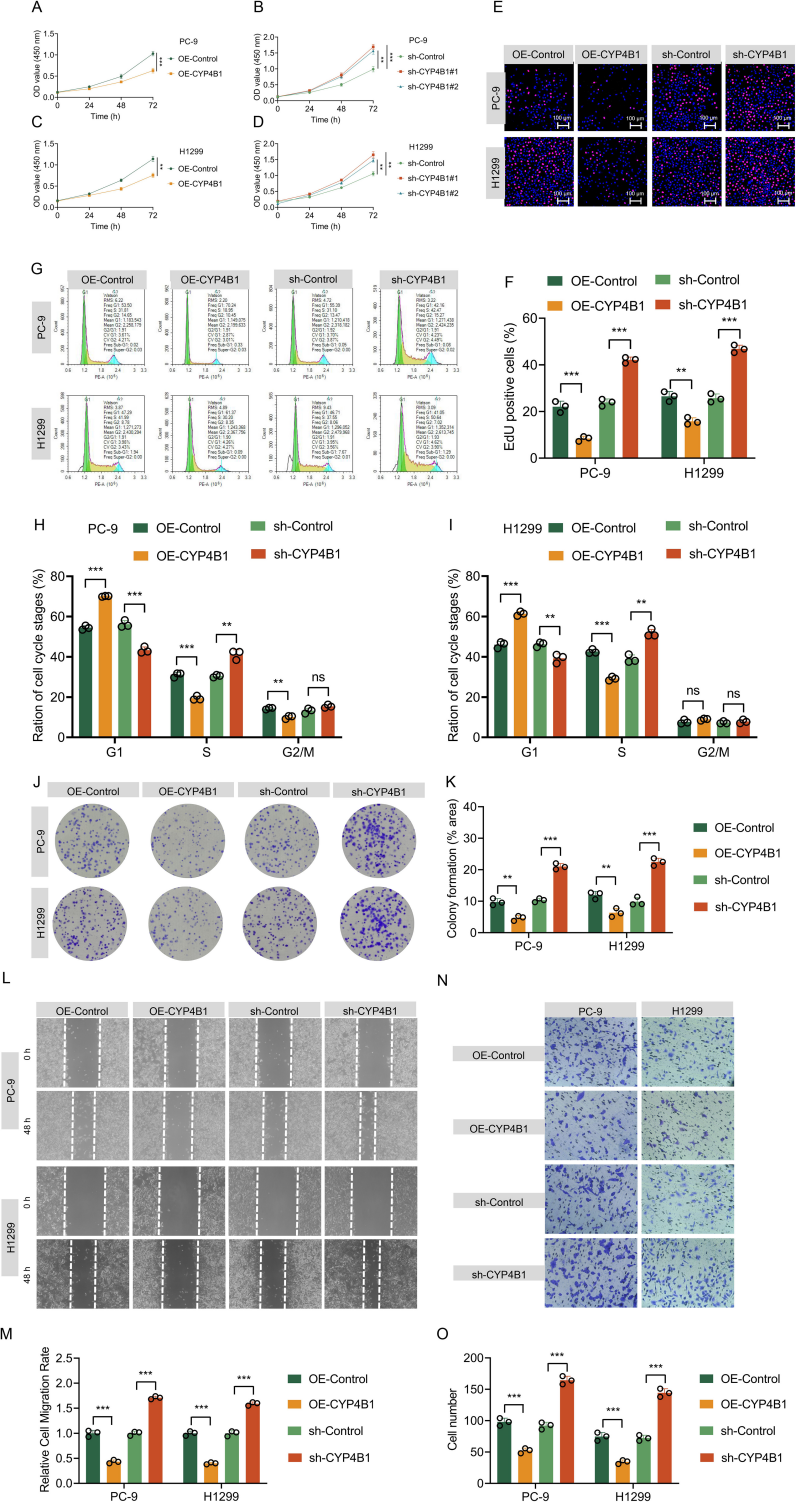
CYP4B1 inhibits the proliferation of LUAD cells *in vitro.***(A–D)** CCK-8 assay. CYP4B1 overexpression significantly reduced the viability of PC-9 and H1299 cells, whereas CYP4B1 knockdown (using two independent shRNA sequences) exerted the opposite effect (n = 4 biological replicates). Subsequent experiments were all performed using sh-CYP4B1#1. **(E, F)** EdU incorporation assay. CYP4B1 overexpression significantly decreased the percentage of EdU-positive cells in PC-9 and H1299 cells, whereas knockdown significantly increased this proportion. **(E)** Representative images; **(F)** Quantitative analysis (n = 3 biological replicates). **(G–I)** Flow cytometry analysis of cell cycle distribution. CYP4B1 overexpression increased the G1-phase proportion and reduced S-phase accumulation in PC-9 and H1299 cells, whereas CYP4B1 knockdown had the opposite effect. **(G)** Representative flow plots; **(H, I)** Quantitative analysis (n = 3 biological replicates). **(J, K)** Colony formation assay. CYP4B1 overexpression inhibited colony formation by PC-9 and H1299 cells, whereas CYP4B1 knockdown promoted colony formation. **(J)** Representative images; **(K)** Quantitative analysis (n = 3 biological replicates). **(L, M)** Wound healing assay. After CYP4B1 overexpression, the migratory capacity of both PC-9 and H1299 cells was significantly decreased, while after CYP4B1 knockdown, the migratory capacity of both cell lines was significantly increased. **(L)** Representative images; M: Quantitative analysis (n = 3 biological replicates). **(N, O)** Transwell assay. After CYP4B1 overexpression, the invasive capacity of both PC-9 and H1299 cells was significantly decreased, while after CYP4B1 knockdown, the invasive capacity of both cell lines was significantly increased. **(N)** Representative images; **(O)** Quantitative analysis (n = 3 biological replicates). **, *P* < 0.01; ***, *P* < 0.001.

To systematically validate the tumor-suppressive role of CYP4B1 in LUAD, we first established stable cell line models: CYP4B1-overexpressing (OE-CYP4B1) and CYP4B1-knockdown (sh-CYP4B1) PC-9/H1299 cell lines were constructed via lentiviral infection. WB analysis confirmed the successful establishment of these stable cell lines (**Figures 2A, B**), and immunofluorescence staining further validated the effective modulation of CYP4B1 protein levels (**Figures 2C, D**). To evaluate the impact of altered CYP4B1 expression on cellular functions, we performed a series of *in vitro* assays. CCK-8 assays showed that CYP4B1 overexpression significantly inhibited the proliferation of both cell lines (**Figures 3A, C**), whereas CYP4B1 knockdown (using two distinct targeting sequences) exerted the opposite effect (**Figures 3B, D**). EdU incorporation assays confirmed these findings, with CYP4B1 overexpression reducing the proportion of EdU-positive cells from 22.1 ± 2.4% to 8.4 ± 1.1% in PC-9 cells and from 26.5 ± 2.1% to 15.8 ± 1.5% in H1299 cells, while CYP4B1 silencing increased the EdU-positive rate to 42.1 ± 1.2% (vs. 23.9 ± 1.5% in controls) and 46.9 ± 1.6% (vs. 25.7 ± 1.8% in controls), respectively (**Figures 3E, F**). Mechanistic exploration via cell cycle analysis revealed that CYP4B1-overexpressing cells exhibited increased G1 phase arrest (PC-9: 70.2 ± 0.2% vs. 54.5 ± 1.0% in controls; H1299: 61.4 ± 1.1% vs. 46.1 ± 1.6% in controls) and reduced S phase proportion (PC-9: 19.5 ± 1.1% vs. 31.1 ± 1.2% in controls; H1299: 29.2 ± 0.9% vs. 42.7 ± 1.0% in controls), whereas CYP4B1 knockdown resulted in the opposite trends (**Figures 3G–I**). The results of the colony formation assay showed that the proliferative capacity of PC-9 and H1299 cells decreased by approximately 52.3% and 46.9% respectively after CYP4B1 overexpression, while it increased by approximately 103.5% and 128.2% respectively after CYP4B1 knockdown (**Figures 3J, K**). The results of the wound healing assay showed that the migratory capacity of PC-9 and H1299 cells decreased by approximately 56.7% and 60% respectively after CYP4B1 overexpression, while it increased by approximately 71% and 59% respectively after CYP4B1 knockdown (**Figures 3L, M**). Transwell assay indicated that the invasive capacity of PC-9 and H1299 cells decreased by approximately 46.7% and 55.6% respectively after CYP4B1 overexpression, while it increased by approximately 78.6% and 97.8% respectively after CYP4B1 knockdown (**Figures 3N, O**).

**Figure 4:**
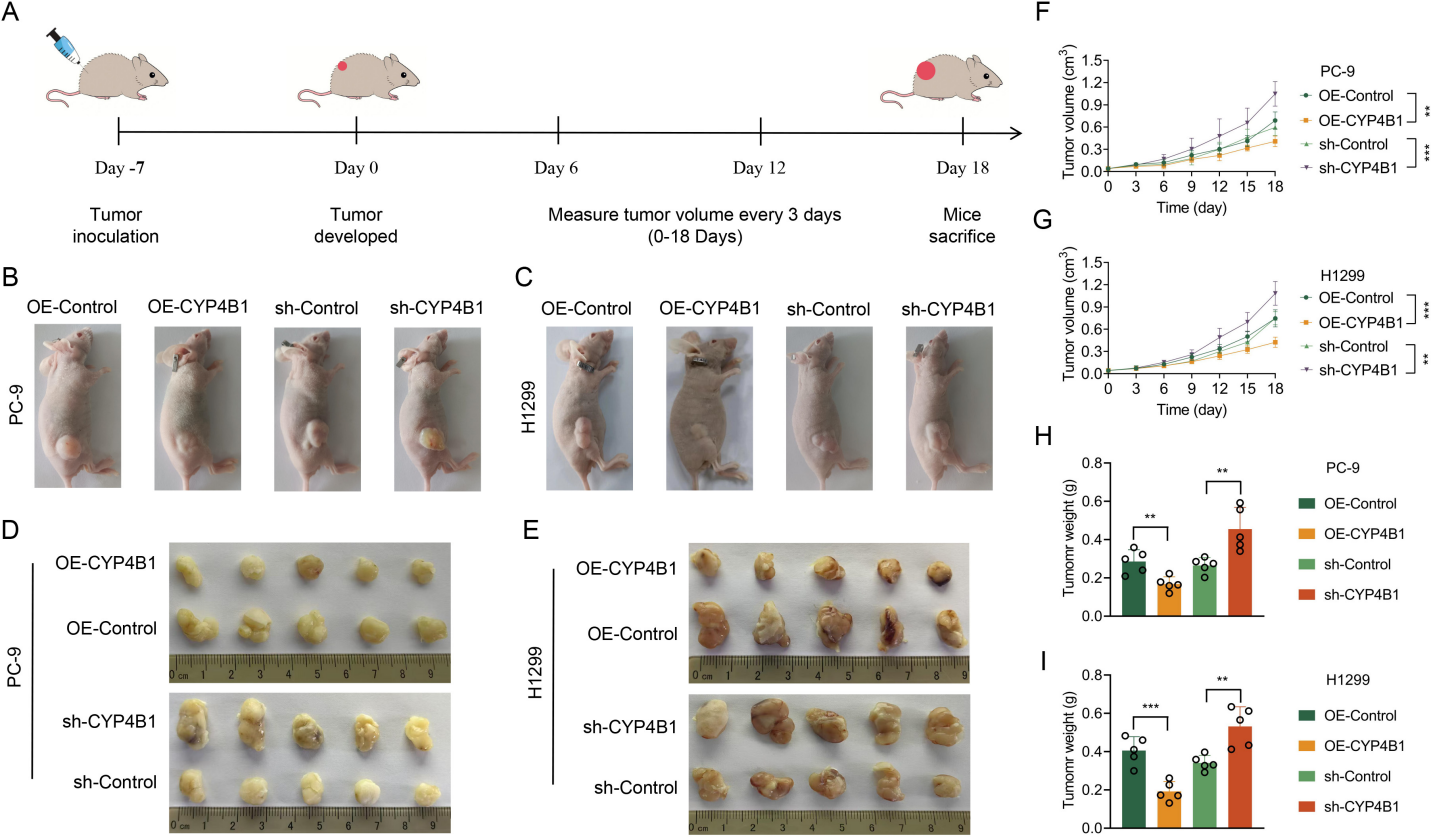
CYP4B1 inhibits the proliferation of LUAD cells *in vivo.***(A)** Schematic of LPC9/H1299-derived xenograft model establishment in BALB/c nude mice (subcutaneous implantation; n = 5 mice per group). **(B, C)** Representative images of mice on day 25 post PC-9/H1299 cells inoculation. **(D, E)** Mice were euthanized 25 days after being inoculated with PC-9/H1299 cells, and the tumor tissues were harvested and photographed. **(F, G)** Mice were measured for tumor size starting from day 7 after inoculation with PC-9/H1299 cells, with measurements taken every 3 days, and tumor growth curves were plotted. **(H, I)** Tumor tissues were harvested and weighed after the mice were sacrificed. *, *P* < 0.05; **, *P* < 0.01; ***, *P* < 0.001.

To translate these *in vitro* findings to an *in vivo* context, we established PC-9 and H1299 xenograft tumor models in nude mice, monitored tumor growth longitudinally, and after euthanizing the animals, subjected the excised tumors to photographic documentation and gravimetric assessment (**Figure 4A**). By day 25 post-implantation, tumors in the OE-CYP4B1 groups were significantly smaller in both volume and weight compared to the OE-Control groups (**Figures 4B–E**): in the PC-9 model, tumor volumes were 0.689 ± 0.103 cm³ vs. 0.408 ± 0.065 cm³ for OE-control vs. OE-CYP4B1 subgroups and 0.595 ± 0.098 cm³ vs. 1.048 ± 0.148 cm³ for sh-control vs. sh-CYP4B1 subgroups (**Figure 4F**), while tumor weights were 0.2869 ± 0.054 g vs. 0.169 ± 0.033 g for OE-control vs. OE-CYP4B1 subgroups and 0.265 ± 0.036 g vs. 0.455 ± 0.101 g for sh-control vs. sh-CYP4B1 subgroups (**Figure 4H**); in the H1299 model, tumor volumes were 0.747 ± 0.103 cm³ vs. 0.425 ± 0.062 cm³ for OE-control vs. OE-CYP4B1 subgroups and 0.748 ± 0.08 cm³ vs. 1.083 ± 0.143 cm³ for sh-control vs. sh-CYP4B1 subgroups (**Figure 4G**), while tumor weights were 0.406 ± 0.065 g vs. 0.192 ± 0.046 g for OE-control vs. OE-CYP4B1 subgroups and 0.341 ± 0.035 g vs. 0.532 ± 0.092 g for sh-control vs. sh-CYP4B1 subgroups (**Figure 4I**).

To reveal the molecular mechanism by which CYP4B1 inhibits the proliferation of LUAD cells, we conducted a series of experiments. Firstly, the results of WB experiments confirmed that overexpression of CYP4B1 could significantly inhibit the phosphorylation of PI3K, AKT, and mTOR in PC-9 and H1299 cells, while the PI3K/AKT/mTOR signaling pathway was abnormally activated after CYP4B1 silencing (**Figures 2A, B**). This result was also confirmed by cellular immunofluorescence in both cell lines (**Figures 2C, D**). In addition, the results of IHC detection showed that CYP4B1 could inhibit the PI3K/AKT/mTOR signaling pathway in clinical LUAD samples (**Figure 2E**). To further confirm that CYP4B1 exerts its antitumor effect by inhibiting the PI3K signaling pathway, we treated CYP4B1-overexpressing H1299 and PC9 cells with 740 Y-P (10 μM), a PI3K agonist. The results showed that 740 Y-P effectively reversed the decreased cell proliferation induced by CYP4B1 overexpression (**Supplementary Figure 4**). Based on the above experimental results, we propose that CYP4B1 inhibits the proliferation of LUAD cells, potentially through suppressing the PI3K/AKT/mTOR signaling pathway.

### 3.3 NFIA acts as a key upstream regulator of CYP4B1 through direct transcriptional activation

**Figure 5:**
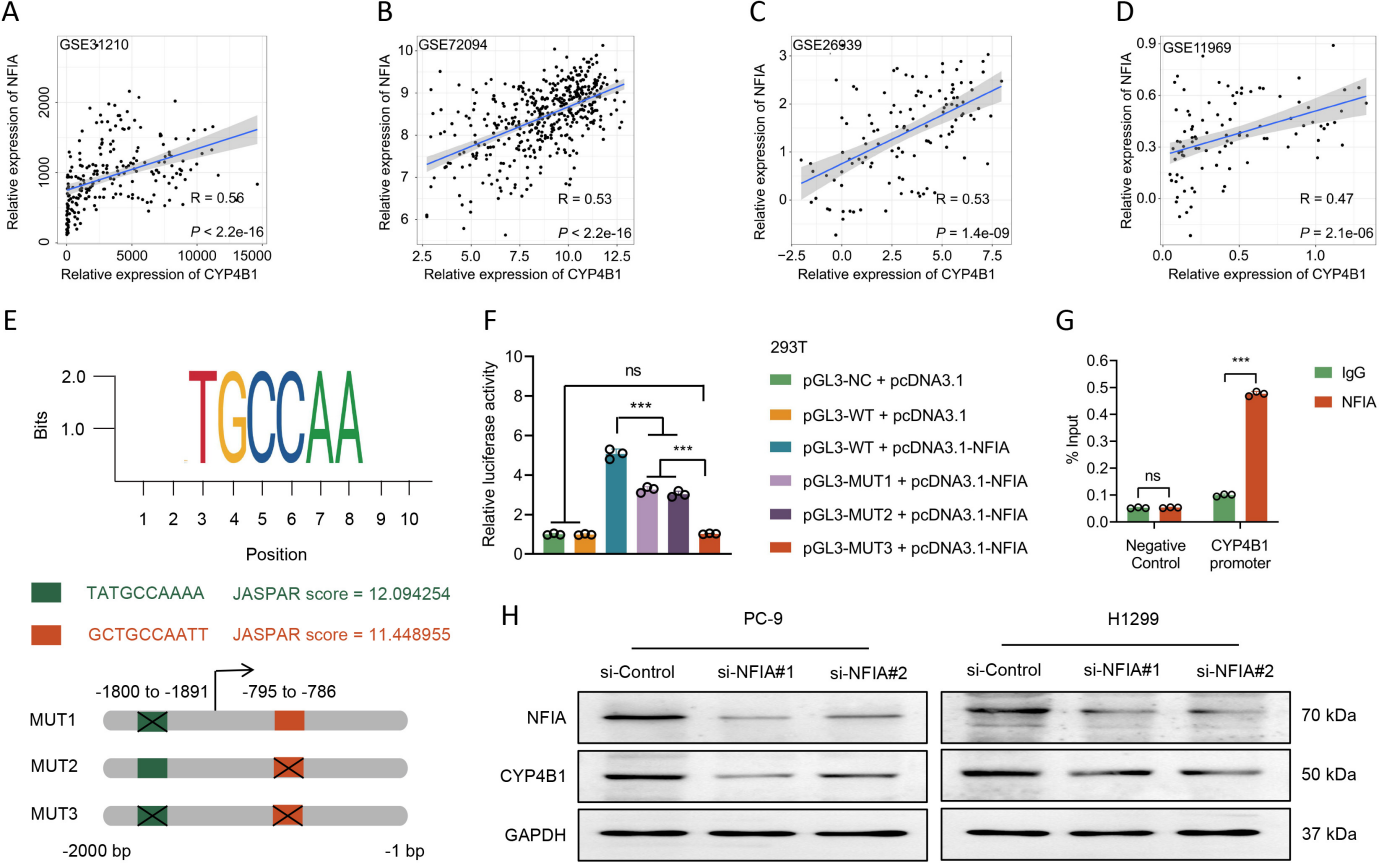
NFIA drives CYP4B1 transcription in LUAD cells. **(A–D)** mRNA-level correlation analysis between NFIA and CYP4B1 in GSE31210, GSE72094, GSE26939, and GSE11969 datasets. **(E)** Schematic representation of wild-type (WT) and mutant (MUT) CYP4B1 promoter sequences with disrupted NFIA binding sites. **(F)** Luciferase reporter assay: 293T cells were co-transfected with pGL3 vectors containing WT or MUT CYP4B1 promoters, pcDNA3.1 or pcDNA3.1-NFIA, and pRL-TK vector (Renilla luciferase internal control). **(G)** ChIP assay confirming direct NFIA binding to the CYP4B1 promoter -795 to -786 bp region. **(H)** siRNA-mediated NFIA knockdown in PC-9 and H1299 cells followed by WB analysis of CYP4B1 protein expression.

To investigate the regulatory mechanisms governing CYP4B1 expression in LUAD, we performed co-expression analyses using public datasets (GSE31210, GSE72094, GSE26939, and GSE11969). These analyses revealed a significant positive correlation between CYP4B1 and NFIA at the mRNA level (**Figures 5A–D**). Additionally, using JASPAR database (http://jaspar.genereg.net/) we identified two putative NFIA binding sites within the CYP4B1 transcriptional regulatory region: a distal site (-1764 to -1755 bp) and a proximal site (-759 to -750 bp) (**Figure 5E**). Collectively, these findings implicate NFIA as a potential upstream regulator of CYP4B1 in LUAD. Dual-luciferase reporter assays demonstrated that co-transfection with an NFIA expression vector enhanced wild-type CYP4B1 promoter activity by 4.07-fold (**Figure 5F**; *P* < 0.001). Mutagenesis of both predicted binding sites abolished this activation, resulting in a 79.7% reduction in luciferase activity compared to wild-type controls (**Figure 5F**; *P* < 0.001). Discrete mutation of the distal and proximal sites reduced activity by 35.5% and 40.2% respectively (**Figure 5F**; *P* < 0.001), indicating both sites contribute to NFIA-mediated transactivation with the proximal site playing a dominant role. ChIP assays in PC-9 cells confirmed direct physical interaction between NFIA and the CYP4B1 promoter, showing a 3.77-fold enrichment of NFIA binding at the -759 to -750 bp region upon NFIA overexpression (**Figure 5G**; *P* < 0.001). Consistent with these results, siRNA-mediated knockdown of NFIA in PC-9 and H1299 cells significantly reduced CYP4B1 protein levels as detected by WB (**Figure 5H**). In conclusion, our integrated bioinformatics and functional analyses establish that NFIA directly binds the CYP4B1 promoter to enhance its transcriptional activity. This NFIA/CYP4B1 axis represents a potential therapeutic target in LUAD and highlights the critical role of transcriptional networks in LUAD pathogenesis.

### 3.4 Construction and validation of CYP4B1-related risk score

**Figure 6:**
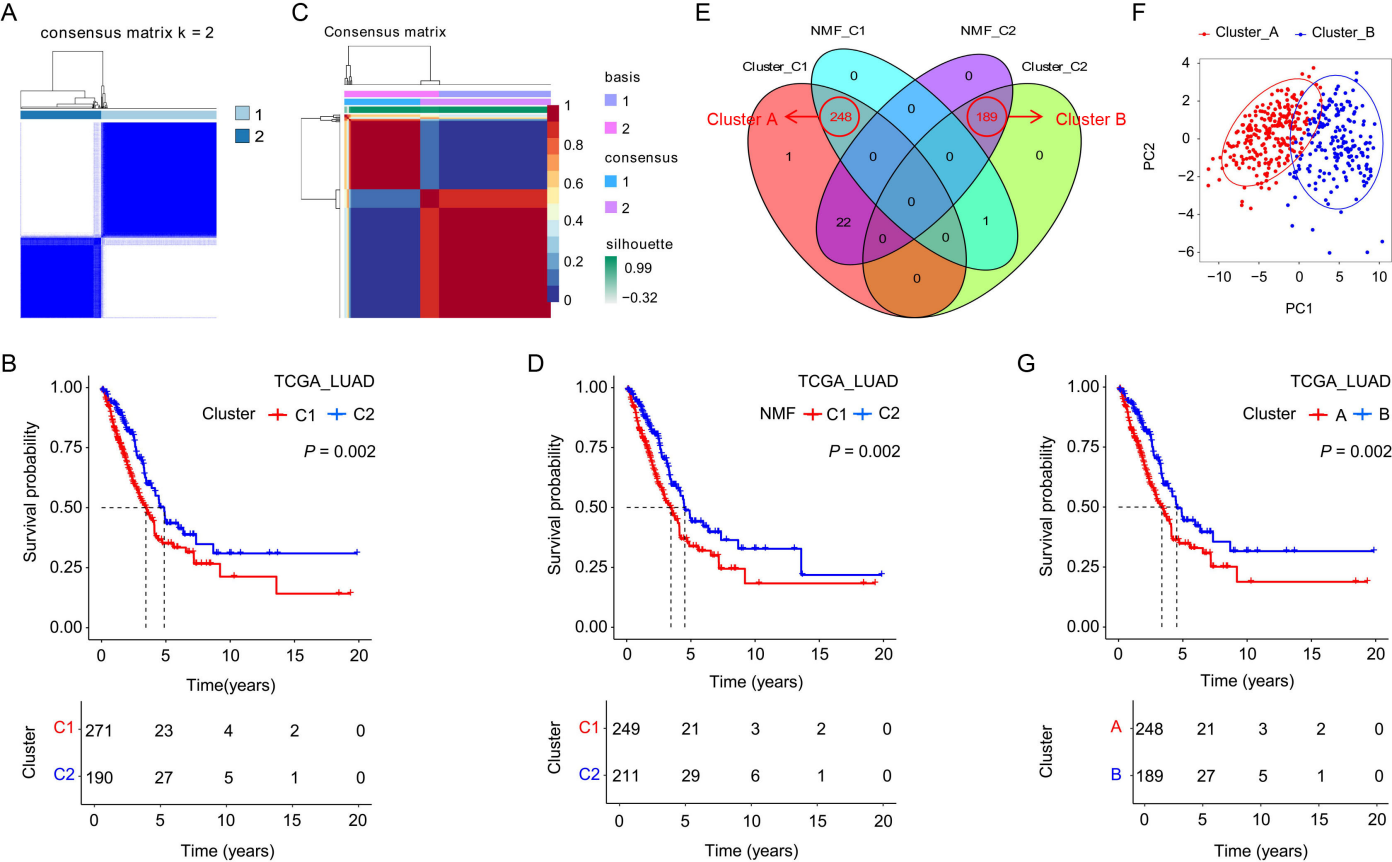
Identification of CYP4B1-related subtypes in LUAD. **(A)** Heatmap of consensus clustering (k = 2) for patients in the TCGA_LUAD dataset. **(B)** KM analysis showed that patients in the C2 group had a longer OS than those in the C1 group. **(C)** The classification of patients from the TCGA_LUAD dataset using NMF, with rank = 2. **(D)** Survival curves of C1 and C2 groups in theTCGA_LUAD dataset. **(E)** The Venn diagram illustrates the grouping method of patients in cluster (A) and (B) **(F)** PCA analysis demonstrated the distinct separation between patients in cluster (A) and (B) **(G)** KM analysis showed that patients in cluster B had a better prognosis.

**Figure 7:**
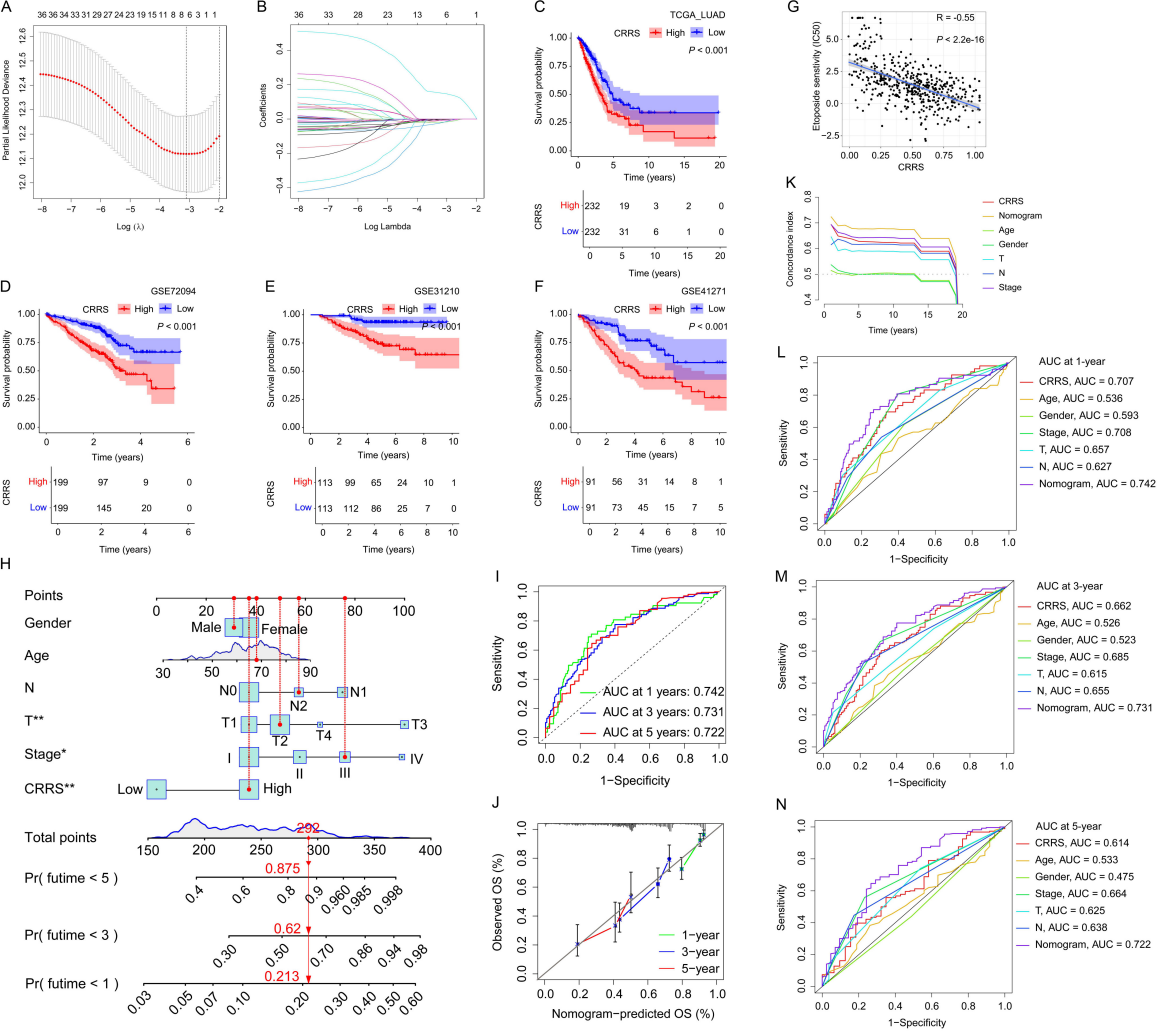
Construction and validation of CYP4B1-related risk score. **(A, B)** Lasso Cox analysis pinpointed 7 core genes most strongly associated with overall survival (OS) in the TCGA_LUAD cohort. **(C–F)** KM curves compared OS differences between high- and low-risk subgroups across the TCGA_LUAD, GSE72094, GSE31210, and GSE41271 cohorts. **(G)** In the TCGA_LUAD cohort, a significant negative correlation was observed between the CRRS and sensitivity to BI-2536. **(H)** A nomogram integrating CRRS, age, gender, tumor grade, T stage, and N stage was constructed to quantify patient prognosis at 1, 3, and 5 years. **(I)** Time-dependent ROC curves assessed the nomogram’s reliability in predicting LUAD patient prognosis at 1, 3, and 5 years. **(J)** Calibration curves evaluated the consistency between nomogram-predicted and observed survival outcomes. **(K)** The C-index is a time-dependent dynamic concordance index. This dynamic C-index plot visually demonstrates the temporal stability of the model’s predictive performance. A consistently high and flat curve indicates that the nomogram maintains robust predictive capability across all time points. **(L–N)** Time-dependent ROC curves highlighted the nomogram’s superiority over other indicators in predicting LUAD patient prognosis 1, 3, and 5 years.

In this study, the TCGA_LUAD cohort served as the training set for developing the CYP4B1-related risk score (CRRS), whose effectiveness and universality were subsequently validated using three external datasets (GSE72094, GSE41271, and GSE31210). From the TCGA_LUAD dataset, we identified 42 genes co-expressed with CYP4B1 (**Supplementary Table 1**; correlation coefficient 0.5; *P* ≤ 0.001). Leveraging the expression profiles of CYP4B1 and these co-expressed genes, we applied consensus clustering and nonnegative matrix factorization (NMF) to stratify patients in the TCGA_LUAD dataset. Through analyzing the consensus matrix for K values from 2 to 10 and the consensus clustering cumulative distribution function (CDF) plot, we determined that K = 2 was the optimal parameter for the consensus clustering method, which classified 271 and 190 patients into C1 and C2 clusters, respectively, with patients in cluster C2 showing a longer overall survival (OS) as indicated by Kaplan–Meier (KM) analysis (**Figures 6A, B**; **Supplementary Figure 5A**). Similarly, based on the cophenetic coefficient and the consensus matrix for ranks ranging from 2 to 10, we found that a rank of 2 was the most suitable number of subgroups for the NMF method, and this approach divided 211 patients into group C2, which also demonstrated a longer OS (**Figures 6C, D**; **Supplementary Figure 5B**). By focusing on the patients in the intersection of the two grouping methods for further analysis, we assigned 248 patients to cluster A and 189 patients to cluster B (**Figure 6E**). Principal component analysis (PCA) results revealed that patients in cluster A and cluster B could be effectively differentiated (**Figure 6F**), and KM analysis further indicated that patients in cluster B had a longer OS (**Figure 6G**). We identified 38 differentially expressed genes (DEGs) significantly associated with patient prognosis from patients in cluster A and B (**Supplementary Table 2**; |logFC| ≥ 2; Adjusted *P* value ≤ 0.01). Incorporating these 38 DEGs into the LASSO-Cox regression model, 7 core genes and their coefficients were selected to develop the CRRS (**Figures 7A, B**), calculated as Score = (0.149 × ANLN) + (-0.002 × C4BPA) + (-0.004 × CRTAC1) + (-0.018 × CYP4B1) + (-0.018 × IRX2) + (-0.003 × NAPSA) + (-0.008 × SFTPB). Patients in both the training and validation sets were divided into high- and low-risk groups based on the median CRRS, with KM analysis showing significantly longer OS for low-risk patients in both sets (**Figures 7C–F**). Additionally, we note that higher CRRS (indicative of a worse prognosis) correlates with predicted resistance to etoposide and paclitaxel—two chemotherapeutic agents commonly used in lung cancer treatment regimens (**Figure 7G**; **Supplementary Figure 6**; |R| ≥ 0.5; *P* ≤ 0.001), validating CRRS as an effective tool for evaluating LUAD prognosis and guiding personalized cancer therapy. The negative correlation suggests that patients in the high-risk group, characterized by lower CYP4B1 expression and a more aggressive tumor phenotype (**Supplementary Figure 7**), may be less sensitive to these agents, possibly due to enhanced survival signaling or drug efflux mechanisms. It is crucial to emphasize that these are computational predictions based on genomic data, and their clinical utility requires prospective validation in pharmacokinetic and clinical response studies. We performed Kaplan-Meier survival analysis stratified by clinical stage (Stage I-II vs. Stage III-IV) within the TCGA cohort. CRRS effectively stratifies patients into high- and low-risk groups with significant survival differences within both early-stage and late-stage subgroups, underscoring its potential clinical applicability across different disease severities (**Supplementary Figure 8**). A nomogram integrating CRRS, age, gender, tumor grade, T stage, and N stage was developed (**Figure 7H**). ROC analysis demonstrated AUC values > 0.72 for 1-, 3-, and 5-year OS predictions (**Figure 7I**), while calibration curves showed strong agreement between predicted and observed outcomes (**Figure 7J**). With superior performance indicated by the C-index and ROC curves compared to other indicators (**Figures 7K–N**), the nomogram emerged as an excellent quantitative tool for assessing LUAD prognosis.

## 4 Discussion

In this study, we systematically delineated the tumor-suppressive role of CYP4B1 in LUAD pathogenesis. We identified its frequent downregulation in tumor tissues, established its functional contribution to inhibiting proliferation both *in vitro* and *in vivo*, elucidated a novel transcriptional regulatory mechanism mediated by NFIA, and uncovered its mechanistic link to the suppression of the PI3K/AKT/mTOR pathway, which had not been previously reported. Furthermore, we translated these mechanistic insights into a clinically applicable CYP4B1-Related Risk Score (CRRS) and nomogram, demonstrating robust prognostic predictive capability across multiple independent cohorts.

Our integrative multi-omics analyses, encompassing six independent datasets, consistently revealed a significant downregulation of CYP4B1 at the mRNA level in LUAD tissues. This finding was corroborated at the protein level in our clinical cohort, aligning with previous reports of reduced CYP4B1 expression in LUAD (19, 30). The consistent loss of CYP4B1 across multiple cohorts solidifies its status as a molecular hallmark of LUAD. Notably, the strong diagnostic power of CYP4B1 (AUC > 0.79) for distinguishing LUAD from normal tissues, coupled with its significant correlation with favorable patient survival in multiple independent cohorts, positions it as a promising and clinically actionable prognostic biomarker.

The tumor-suppressive function of CYP4B1 was unequivocally demonstrated through a series of functional experiments. Modulating CYP4B1 expression in LUAD cell lines revealed its potent capacity to inhibit cell proliferation, induce G1/S cell cycle arrest, and suppress colony formation. These *in vitro* findings were further validated in xenograft models, where CYP4B1 overexpression significantly curtailed tumor growth. Mechanistically, we reveal the oncogenic PI3K/AKT/mTOR signaling pathway is a potential downstream effector of CYP4B1. A battery of assays, including Western blot, immunofluorescence, and IHC, consistently showed that CYP4B1 overexpression suppresses the phosphorylation of PI3K, AKT, and mTOR, whereas its knockdown has the opposite effect. Given the well-established role of the PI3K/AKT/mTOR axis in promoting cell survival and proliferation (31, 32), its inhibition provides a plausible molecular explanation for the anti-proliferative effects of CYP4B1 (33, 34). It is important to note that while our data show a consistent inverse relationship between CYP4B1 expression and PI3K/AKT/mTOR pathway activation, the experimental design does not establish a direct causal link. Future studies employing pharmacological rescue experiments are warranted to conclusively determine if modulating this pathway can reverse the phenotypic effects of CYP4B1 alteration. Our discovery that CYP4B1 loss is correlated with hyperactivation of the oncogenic PI3K/AKT/mTOR pathway provides a plausible mechanistic hypothesis to explain its tumor-suppressive function and pinpoints a specific signaling axis for future functional validation.

An intriguing aspect of CYP4B1 biology is its context-dependent role in cancer. While our data firmly support its tumor-suppressive function in LUAD, other studies, such as those in bladder cancer, report CYP4B1 as a potential oncogene by activating procarcinogens (17). This apparent paradox may stem from tissue-specific expression patterns, differential substrate availability, or distinct microenvironments. The high basal expression of CYP4B1 in the lung suggests that its loss may be particularly detrimental in this context, potentially disrupting crucial homeostatic processes. It is important to consider that CYP4B1, as a key enzyme in fatty acid ω-hydroxylation and a producer of signaling molecules like 20-HETE, is poised to influence a broader biological network beyond a single signaling pathway (35–38). Its tumor-suppressive effects may therefore also involve the modulation of lipid metabolism, redox homeostasis, or the tumor immune microenvironment, which represent compelling avenues for future investigation.

To unravel the upstream events leading to CYP4B1 downregulation in LUAD, we investigated its transcriptional regulation. We demonstrated a significant positive correlation between NFIA and CYP4B1 mRNA levels, confirmed NFIA’s binding to specific sites on the CYP4B1 promoter via ChIP assay, and established its role in transactivating the CYP4B1 promoter through luciferase reporter assays. The dependence of CYP4B1 protein expression on NFIA levels further solidifies this regulatory link. Our integrated bioinformatics and functional analyses identified NFIA as a key upstream regulator of CYP4B1, demonstrating its direct binding and transactivation of the CYP4B1 promoter. This finding aligns with and extends previous reports of NFIA’s tumor-suppressive role in NSCLC, proposing a coherent NFIA-CYP4B1 regulatory axis that contributes to LUAD suppression (21, 22). While we have established NFIA as a direct transcriptional activator of CYP4B1, our study has not functionally demonstrated that the tumor-suppressive effects of NFIA are entirely dependent on CYP4B1. Future studies employing rescue experiments are needed to fully validate this axis as the primary mechanism for NFIA’s function in LUAD. Beyond this transcriptional control, alternative mechanisms such as epigenetic silencing via promoter hypermethylation, regulation by other transcription factors or non-coding RNAs, and post-translational modifications affecting CYP4B1 protein stability could concurrently contribute to its loss of expression. Elucidating these alternative and compensatory pathways will be crucial for a comprehensive understanding of CYP4B1 dysregulation in LUAD and may uncover additional targets for therapeutic intervention.

Capitalizing on the mechanistic role of CYP4B1, we sought to translate our findings into a clinically useful tool. We developed a CRRS based on seven core genes identified through robust bioinformatics pipelines. The CRRS effectively stratified patients into distinct high- and low-risk groups with significantly divergent overall survival across both the training and multiple independent validation cohorts. Its significant correlation with sensitivity to various chemotherapeutic agents underscores its potential for guiding personalized therapy. To enhance clinical applicability, we integrated the CRRS with standard clinical variables into a user-friendly nomogram, which demonstrated superior predictive accuracy for 1-, 3-, and 5-year overall survival compared to individual clinical parameters. This model represents a tangible step towards precision oncology for LUAD patients.

This study has several limitations. First, while we used two LUAD cell lines with distinct genetic backgrounds (EGFR-mutant and p53-null), expanding functional validation to models with other driver mutations (e.g., KRAS) would strengthen the generalizability of our conclusions. Second, the clinical utility of the CRRS and nomogram, though validated in multiple public cohorts, requires prospective validation in large, multi-center, and ethnically diverse populations. Third, our mechanistic exploration centered on the PI3K/AKT/mTOR axis; the potential roles of CYP4B1 in other malignant phenotypes, such as metastasis and therapy resistance, and its interplay with other pathways remain to be fully elucidated. Future work should include comprehensive *in vivo* metastasis models, co-clinical studies, and metabolomic/lipidomic profiling to dissect the precise link between CYP4B1’s enzymatic activity and its tumor-suppressive function.

## Data Availability

The original contributions presented in the study are included in the article/**Supplementary Material**. Further inquiries can be directed to the corresponding authors.
