# Supplementary material for: CYP4B1 inhibits lung adenocarcinoma progression via PI3K/AKT/mTOR pathway: mechanistic insights and development of a CYP4B1-related prognostic signature
Source: Front Oncol. 2026 Jan 26;15:1661650. doi: 10.3389/fonc.2025.1661650 (PMC12883422; doi:10.3389/fonc.2025.1661650)
Supplement: Supplementary file 1 [file DataSheet1.docx]

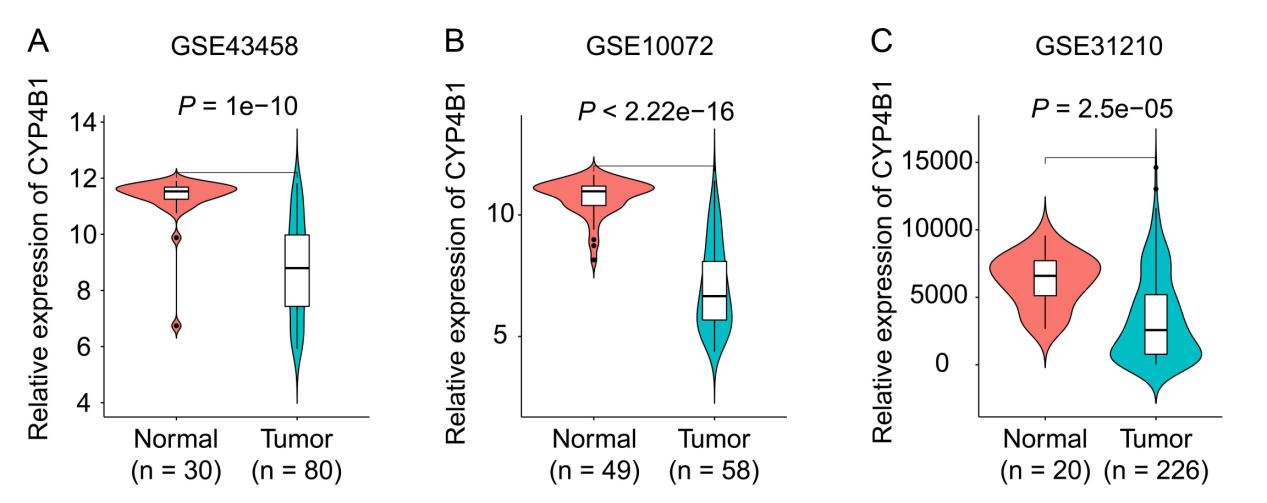


**Supplementary Figure 1. CYP4B1 is lowly expressed in LUAD tissues**

(A-C) CYP4B1 mRNA expression levels in normal vs. LUAD tissues from GSE43458, GSE10072 and GSE31210 datasets.


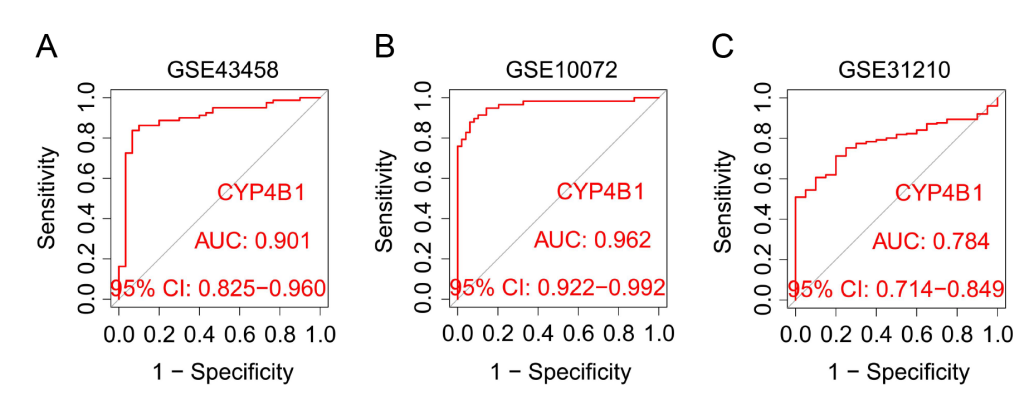


**Supplementary Figure 2. ROC curve based on the expression level of CYP4B1**

(A-C) ROC curve analysis of CYP4B1 expression for distinguishing LUAD from normal tissues in GSE43458, GSE10072 and GSE31210 datasets.


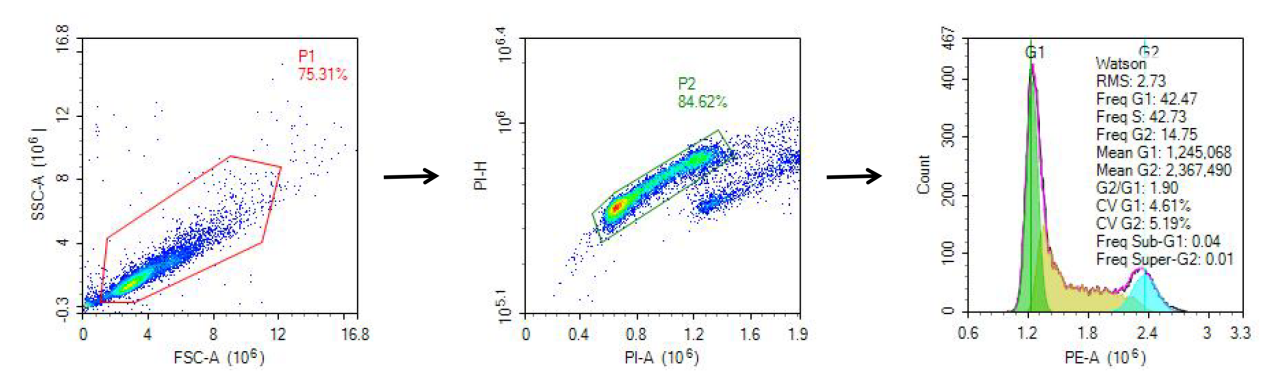


**Supplementary Figure 3. Gating strategy of flow cytometry**


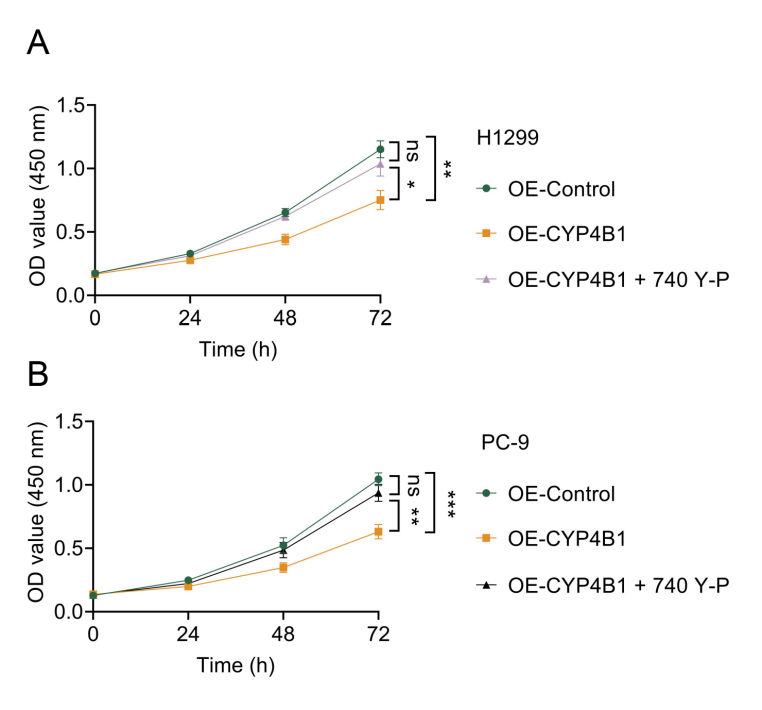


**Supplementary Figure 4. 740 Y-P reverses the inhibitory effect of CYP4B1 overexpression on lung adenocarcinoma cells**

(A-B) **740 Y-P (10 μM) can reverse the inhibitory effect of CYP4B1 overexpression on the proliferation of H1299 and PC-9 cells.**


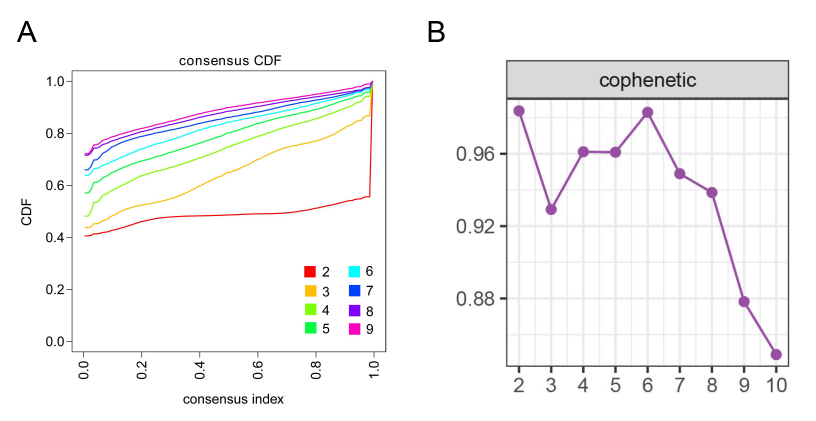


**Supplementary Figure 5. CDF curves of consensus clustering and cophenetic coefficients of NMF**

(A) The CDF curve of consensus clustering, K = 2 to 10. (D) The cophenetic value of **ranks from** 2 to 10 in NMF.


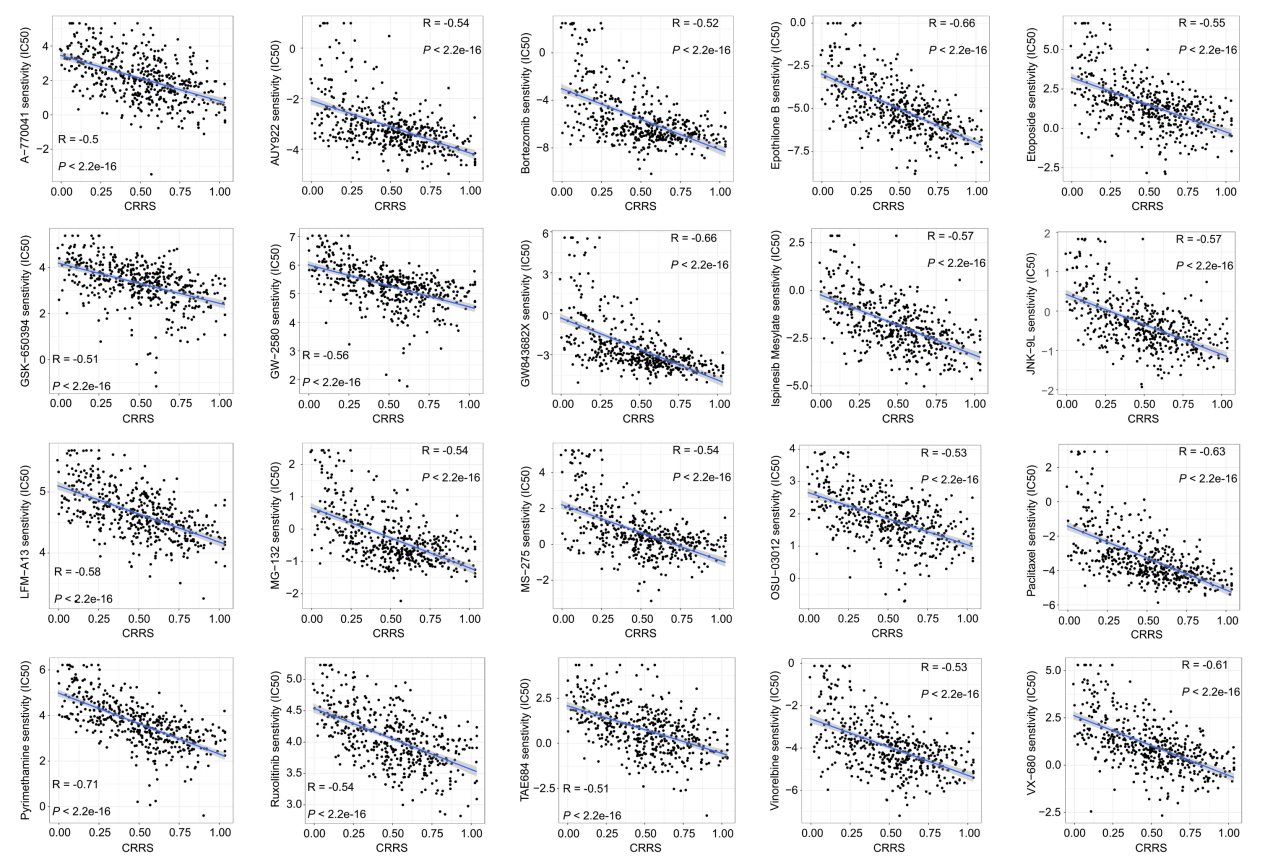


**Supplementary Figure 6. Analysis of the correlation between CRRS and the sensitivity of LUAD patients to clinical drugs**


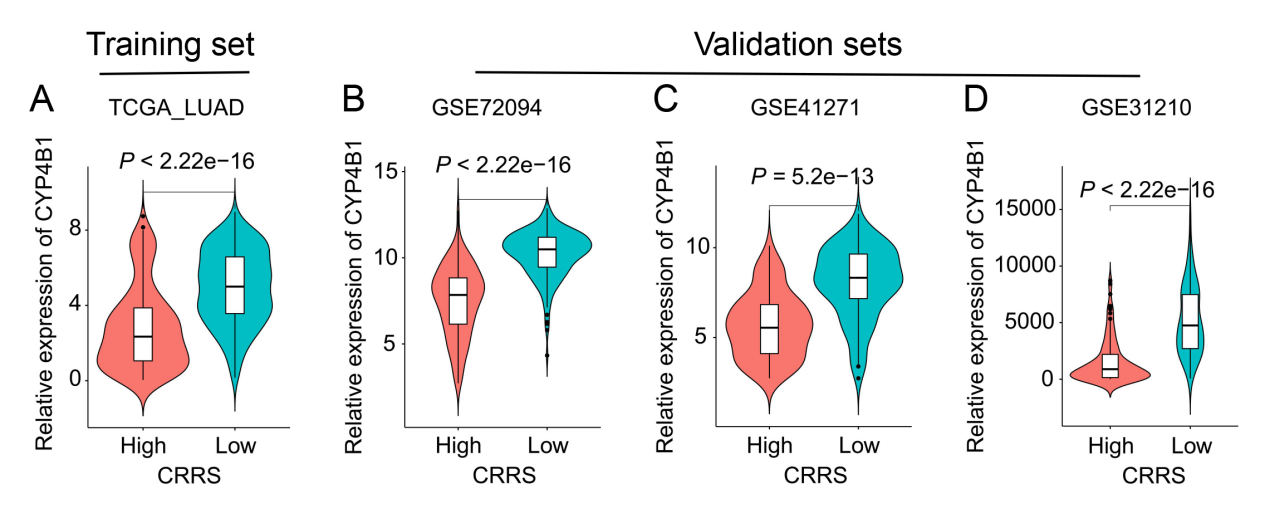


**Supplementary Figure 7. CYP4B1 is lowly expressed in the high-CRRS subgroups**

(A-D) CYP4B1 mRNA expression levels in high-CRRS vs. low-CRRS subgroups from the training set and validation sets.


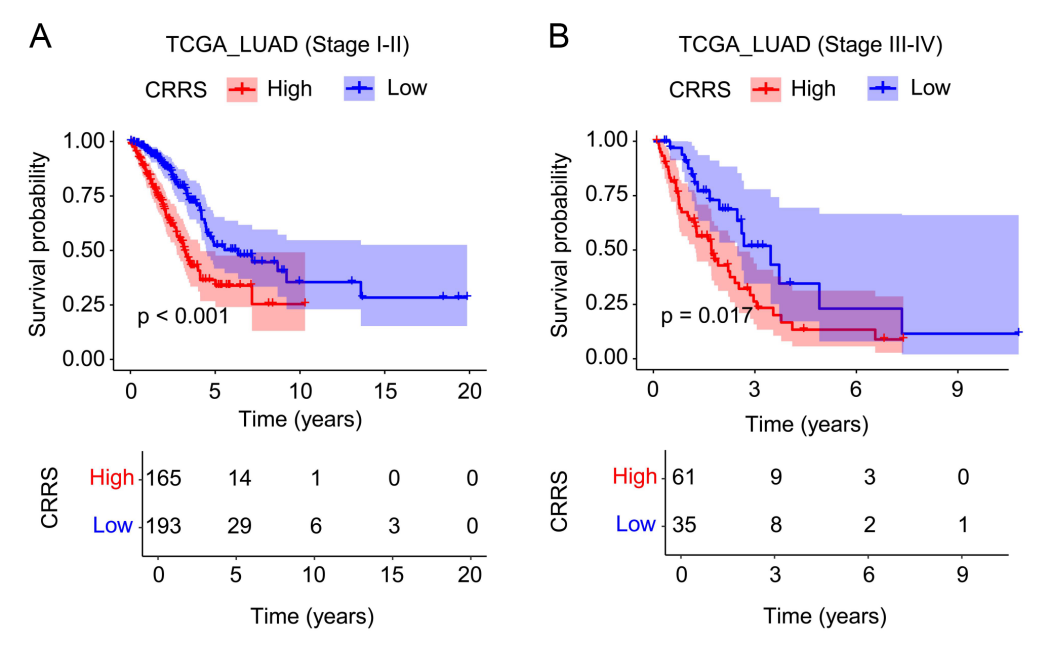


**Supplementary Figure 8. Kaplan-Meier survival analysis stratified by clinical stage within the TCGA cohort**

(A-B) CRRS effectively stratifies patients into high- and low-risk groups with significant survival differences within both early-stage and late-stage subgroups.
